# Incidence and management patterns of alcohol-related liver disease in Korea: a nationwide standard cohort study

**DOI:** 10.1038/s41598-021-86197-z

**Published:** 2021-03-23

**Authors:** Ha Il Kim, Seo Young Park, Hyun Phil Shin

**Affiliations:** 1 Division of Gastroenterology, Department of Internal Medicine, Kyung Hee University Hospital at Gangdong, Kyung Hee University School of Medicine, 892 Dongnam-ro, Gangdong-gu, Seoul, 05278 Korea; 2 Department of Clinical Epidemiology and Biostatistics, Asan Medical Center, Seoul, Korea; 3 Division of Gastroenterology, Department of Internal Medicine, Kyung Hee University School of Medicine, Seoul, Korea

**Keywords:** Hepatology, Health care, Medical research

## Abstract

The recent incidence and management patterns of alcohol-related liver disease (ARLD) are not well defined in Korea. We sought to evaluate the epidemiology of ARLD with regard to disease severity and alcohol cessation management after diagnosis. We performed an observational cohort study of standardized Common Data Model data from the Health Insurance Review and Assessment-National Patient Samples database between 2012 and 2016. The incidence and demographic properties of ARLD were extracted and divided into non-cirrhotic alcoholic liver disease (ALD) and alcoholic liver cirrhosis (ALC). ALC was compared with non-alcoholic cirrhosis by severity at diagnosis. The management patterns were captured by the initiation of pharmaco- and behavioral therapy for alcohol cessation. We analyzed data from 72,556 ALD to 7295 ALC patients. The ALD incidence was stable from 990 to 1025 per 100,000 people. In ALD, the proportion of patients who were ≥ 65 years old, the proportion of female patients, and the comorbidity index increased significantly during the study period (all *P* values < 0.001). ALC accounted for > 20% of all cirrhosis, with decompensation occurring twice as often as in non-alcoholic cirrhosis. The initiation of alcoholism management was stationary in ARLD, remaining at < 10% for both pharmacotherapy and behavioral therapy, regardless of severity or the site of diagnosis. The incidence of ARLD did not decrease during the study period. Moreover, an increasing trend in the proportion of people vulnerable to drinking was observed. Unfortunately, management for the cessation of alcohol use remains very low. The best way to manage ARLD should be evaluated in further study.

## Introduction

Alcoholism is a major contributor to morbidity worldwide^1^. Alcohol-related liver disease (ARLD) accounts for a major portion of alcohol-related disorders^2^. The burden of ARLD varies from country to country, and its severity is consistently associated with alcohol consumption^3–5^. Therefore, in countries with high alcohol consumption, ARLD is an important problem for physicians. However, unlike the increasing concerns about other types of liver disease, such as viral hepatitis and non-alcoholic fatty liver disease, ARLD fails to be considered a priority despite its medical and socioeconomic burden^6–8^.

From a global perspective, Korea is one of the highest alcohol consumers^1,7^. A recent population-based study showed that the prevalence of ARLD had doubled for two decades^9^. Another nationwide survey showed that the amount of alcohol consumption and the rate of high-risk drinking have remained high^10^. Although these recent studies suggest that the actual burden of ARLD might have worsened, only limited information is available to evaluate the incidence of ARLD with or without cirrhosis.

The backbone of treatment for ARLD is the cessation of alcohol use, and the most effective way to control drinking is a combination of pharmacologic and behavioral therapy^11–14^. Various studies have shown that the cessation of alcohol use stops disease progression and improves clinical outcomes^13–15^. Unfortunately, no previous population-level study has captured the pattern of management after an ARLD diagnosis.

Therefore, we evaluated the nationwide incidence of and management patterns for ARLD in Korea. First, we captured the incidence of ARLD compared with other liver-disease etiologies. Second, we evaluated the management patterns for ARLD that encourage the cessation of alcohol use. Using recent nationwide data collected in a common data model (CDM), we explored whether the nationwide burden of ARLD is misunderstood or unmet needs for ARLD management are present in Korea.

## Methods

### Data source

In this study, we used the Health Insurance Review and Assessment-National Patient Samples (HIRA-NPS) database from 2012 to 2016, which had already been converted to the standardized Observational Medical Outcomes Partnership (OMOP)-CDM. HIRA is a repository of claims data collected in the process of reimbursing healthcare providers for services to all citizens in Korea under the universal coverage system^16^. The HIRA-NPS is a 3% sample of national patient records (about 1,000,000) per year that is extracted using a stratified randomized sampling method according to sex and age group at the patient level. It includes about 13% (approximately 700,000) of inpatient records and 1% (approximately 400,000) of outpatient records, including all medical claim and prescriptions data for each year, which are calculated under the assumption of an acceptable sampling error range and the normal distribution to determine the optimal size of the HIRA-NPS.

It is difficult to characterize or analyze data from the HIRA-NPS and hospitals (especially international hospitals) in the same way or using the same tools because healthcare data sets are built using a wide variety of data models and often local terminologies^17–19^. Recently, HIRA-NPS was converted to the OMOP-CDM, which harmonizes disparate coding systems into a standardized vocabulary with minimal information loss^17,18^. Non-standard HIRA-NPS terms were standardized using mapping. HIRA data were initially recorded using International Classification of Diseases version 9 codes, and they were mapped to the Systemized Nomenclature for Medicine-Clinical Terms (SNOMED-CT) codes in the CDM data^17^. The OMOP-CDM version of the HIRA-NPS database is provided as an open source in Korea, which gives it advantages over the original HIRA-NPS database, which requires pre-approval, a usage fee, and limited use within a predetermined platform for on-line or off-line use. This study protocol was approved by the Institutional Review Board of Kyung Hee University in South Korea, all the study methods were performed in accordance with the guidelines and regulations (KHNMC IRB 2020-08-008).

### Study design and cohort definition

We conducted a retrospective, observational cohort study to address two main outcomes: (1) determine the incidence and demographic characteristics of ARLD, (2) compare the incidence and severity of cirrhosis between ARLD and non-ARLD subjects, and (3) determine the management patterns for ARLD. All subjects were ≥ 18 years old between January 1, 2012, and December 31, 2016. The patients in the study cohort were diagnosed with liver disease for the first time in their history with the medical insurance program. We describe the demographic characteristics by index year. The variables used in this study are age, age group (≤ 40 years, 40–65 years, ≥ 65 years), sex, and Charlson comorbidity index (CCI). The algorithm from the National Cancer Institute (NCI) for constructing a comorbidity index was used, including the adaptations suggested by Deyo and Romano to estimate a CCI for all patients^20–22^.

ARLD was divided into two groups, alcoholic liver disease (ALD, non-cirrhotic ARLD) and alcoholic liver cirrhosis (ALC), excluding other etiologies for chronic liver disease. ALD was diagnosed based on the following concept codes without liver cirrhosis: 41,309,000 (alcoholic liver damage), 235,875,008 (alcoholic hepatitis), 235,881,000 (alcoholic hepatic failure), and 50,325,005 (alcoholic fatty liver). ALC was diagnosed based on the following concept codes: 420,054,005 (alcoholic cirrhosis) and 235,880,004 (alcoholic fibrosis and sclerosis of liver).

The features of hepatic decompensation were defined as the presence of hepatic encephalopathy, varices, and ascites without secondary cause^23^. When ARLD was extracted from the CDM database, any diagnoses corresponding to viral and non-viral/non-ARLD were excluded. Diagnoses of non-viral/non-ARLD include non-alcoholic fatty liver disease, non-alcoholic steatohepatitis, and other minor etiologies for chronic liver disease: primary biliary cholangitis, primary sclerosing cholangitis, Wilson’s disease, hemochromatosis, and autoimmune hepatitis. Patients with a previous history of primary liver cancer and liver transplantation recipients were also excluded. Concept identification and the codes for the concept sets used in our study population were classified into categories based on SNOMED-CT codes and are described in Supplementary Table S1.

### Assessment of critical events and patterns of treatment for ARLD

The incidence rate of critical events in ARLD was assessed using three categories of events that occurred within 30 days of diagnosis. The result of ALC was compared to non-alcoholic cirrhosis: (1) visit to the emergency department (ED), (2) need for intensive care unit (ICU) care, and (3) death^24–28^. The need for ICU care was defined as tracheal intubation, continuous renal replacement therapy, or the use of vasopressors. Thirty-day in-hospital or ED death was analyzed using the death domain of the CDM without liver transplantation.

To evaluate the treatment patterns, the initial time points of pharmacological and behavioral therapy were captured within 60 days after a diagnosis of ARLD. Pharmacological therapy was naltrexone or acamprosate (single or combination treatment), which are approved medications to treat chronic alcoholism in Korea^12^. Patients who underwent prior pharmacological treatment or were prescribed any opioids within 60 days of diagnosis were excluded^1^. Behavioral therapy comprised individual psychotherapy, family psychotherapy procedures, and group psychotherapy^29^, which were captured from the study cohort within 60 days of diagnosis.

### Statistical analyses

OHDSI analysis tools are embedded in the interactive analysis platform ATLAS. ATLAS version 2.7.6 was used herein, and we analyzed the platform of FEEDER-NET, a health big-data platform based on the OHDSI-CDM and supported by the Korean National Project^30^. Data for normally distributed continuous variables are presented as the mean ± standard deviation, and data for categorical variables are presented as the number (percentage). The chi-squared test was used to examine relationships between categorical variables, and a 1-way analysis of variance (ANOVA) was used to compare the mean values of continuous variables across the groups. Temporal linear trends in proportions were assessed using the Cochran-Armitage test for trends. All *P* values were two-tailed, and *P* values less than 0.05 were considered statistically significant.

### Ethical approval

This study was approved by the Institutional Review Board of the study institution (KHNMC IRB 2020-08-008).

## Results

### Incidence and demographic characteristics of ARLD

A total of 72,556 patients diagnosed with ALD were captured from the study database between 2012 and 2016: 14,561 in 2012, 14,327 in 2013, 14,061 in 2014, 14,832 in 2015, and 14,775 in 2016. The average age range was 51–54 years. The proportions of patients ≥ 65 years old and female increased significantly during the study period (*P* < 0.001 for both). The CCI also increased significantly during the study period (*P* < 0.001) (Table 1). The annual incidence rates of ALD were 990–1025 per 100,000 people, comprised more than 75% of alcohol-related disorders diagnosed during the study period (Supplementary Table S2).

**Table 1:** Demographic characteristics of the study population with alcoholic liver disease and alcoholic cirrhosis by index year.

Demographic characteristics	2012	2013	2014	2015	2016	*P*
**Alcoholic liver disease**	14,561	14,327	14,061	14,832	14,775	
Proportion per 100,000 people	1025	1002	990	1021	1007	
Age, m ± SD	52.62 ± 12.78	53.07 ± 12.82	51.32 ± 12.64	53.81 ± 13.16	54.30 ± 12.99	< 0.001
Age group, n (%)						< 0.001
≤ 40 year	2492 (17.1)	2324 (16.2)	2713 (19.3)	2287 (15.4)	2087 (14.1)	
≥ 65 year	2641 (18.1)	2684 (18.7)	2127 (15.1)	3067 (20.7)	3134 (21.2)	
Male, n (%)	11,498 (79.0)	11,197 (78.2)	10,925 (77.7)	11,408 (76.9)	11,219 (75.9)	< 0.001
Charlson comorbidity index, m ± SD	1.39 ± 1.95	1.70 ± 1.93	1.75 ± 1.99	1.83 ± 2.00	1.86 ± 1.97	< 0.001
**Alcoholic liver cirrhosis (per total cirrhosis, %)**	1463 (23.8)	1415 (22.9)	1458 (22.0)	1429 (21.2)	1530 (21.1)	
Age, m ± SD	56.78 ± 11.08	57.33 ± 10.94	55.55 ± 11.41	58.00 ± 11.19	57.62 ± 11.02	< 0.001
Age group, n (%)						0.005
≤ 40 year	100 (6.8)	89 (6.3)	120 (8.2)	74 (5.2)	82 (5.4)	
≥ 65 year	365 (24.9)	360 (25.4)	329 (22.6)	396 (27.7)	390 (25.5)	
Male, n (%)	1268 (86.7)	1237 (87.4)	1268 (87.0)	1207 (84.5)	1307 (85.4)	< 0.001
Charlson comorbidity index, m ± SD	3.42 ± 2.45	3.45 ± 2.48	3.50 ± 2.46	3.51 ± 2.46	3.47 ± 2.38	0.113
Decompensation at diagnosis	594	615	592	540	586	

A total of 7295 patients diagnosed with ALC were captured between 2012 and 2016: 1463 in 2012, 1415 in 2013, 1458 in 2014, 1429 in 2015, and 1530 in 2016. The proportion of ALC within all cirrhosis cases ranged from 21.1 to 23.8%, and the mean age and the proportion of females increased significantly during the study period (both *P* < 0.001). Although CCI increased in the ALD group, it remained stable in the ALC group throughout the study period (*P* = 0.113) (Table 1). When comparing average age and CCI, the mean values in the ALC group were significantly higher than those of the ALD group during the study period (all *P* < 0.001). The proportion of females was higher in the ALD group than the ALC group during the study period (*P* < 0.001) (Supplementary Table S3).

**Figure 1:**
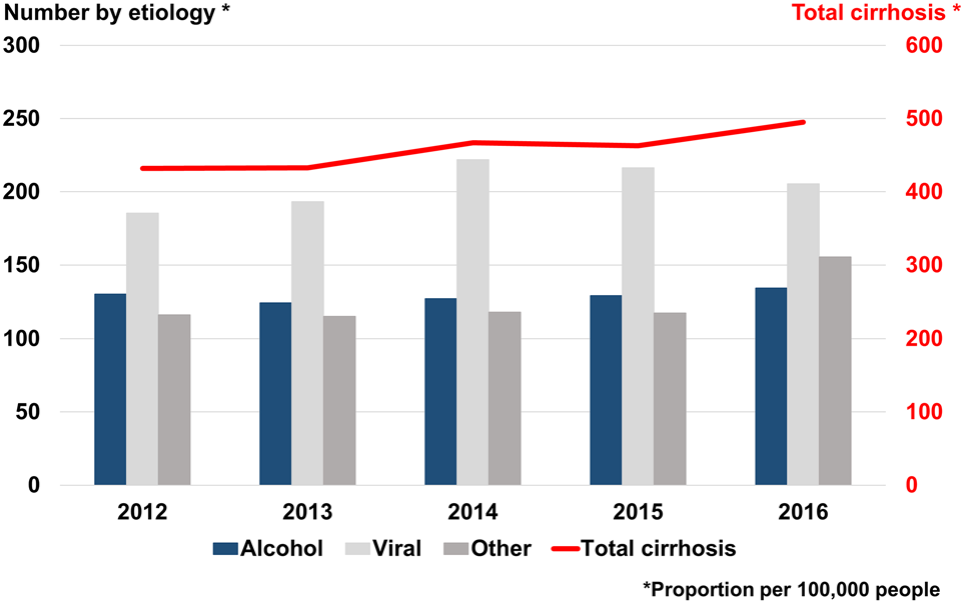
Incidence of cirrhosis per 100,000 patients in the total population.

**Figure 2:**
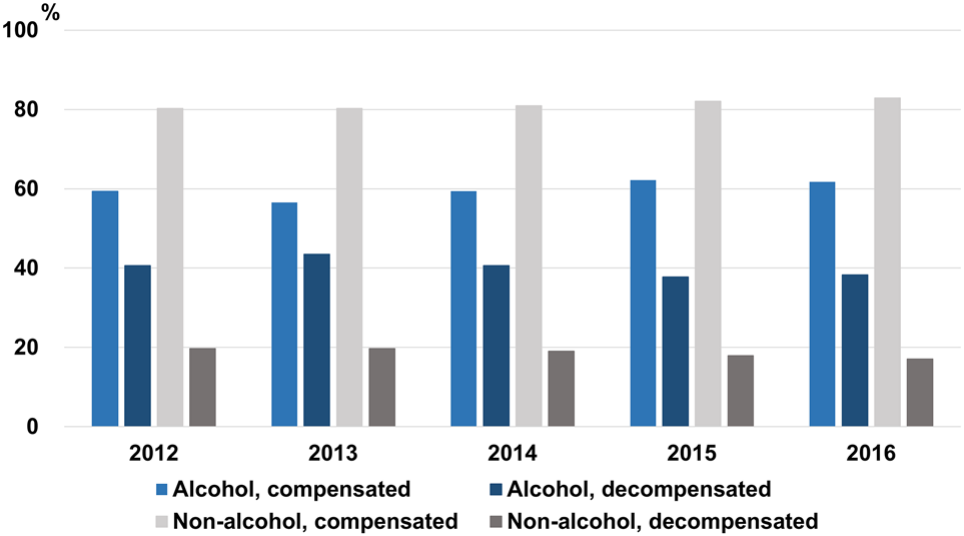
Proportions of non-alcoholic and alcoholic liver cirrhosis at initial diagnosis.

The incidence of ALC remained similar (124–134 per 100,000); in contrast, non-viral/non-alcoholic cirrhosis increased during the study period (Fig. 1). The proportion of ALC patients who had decompensation was 37.8–43.5%, almost double that of non-alcoholic cirrhosis patients (17.1–19.7%) (Fig. 2). During the study period, the ALC group consistently showed a younger mean age, higher CCI, and male predominance compared to the non-alcoholic cirrhosis group (all *P* < 0.001) (Supplementary Table S4). A summary of the demographics for non-alcoholic cirrhosis is presented in Supplementary Table S5.

### Comparison of critical events between alcoholic liver cirrhosis and non-alcoholic liver cirrhosis

**Figure 3:**
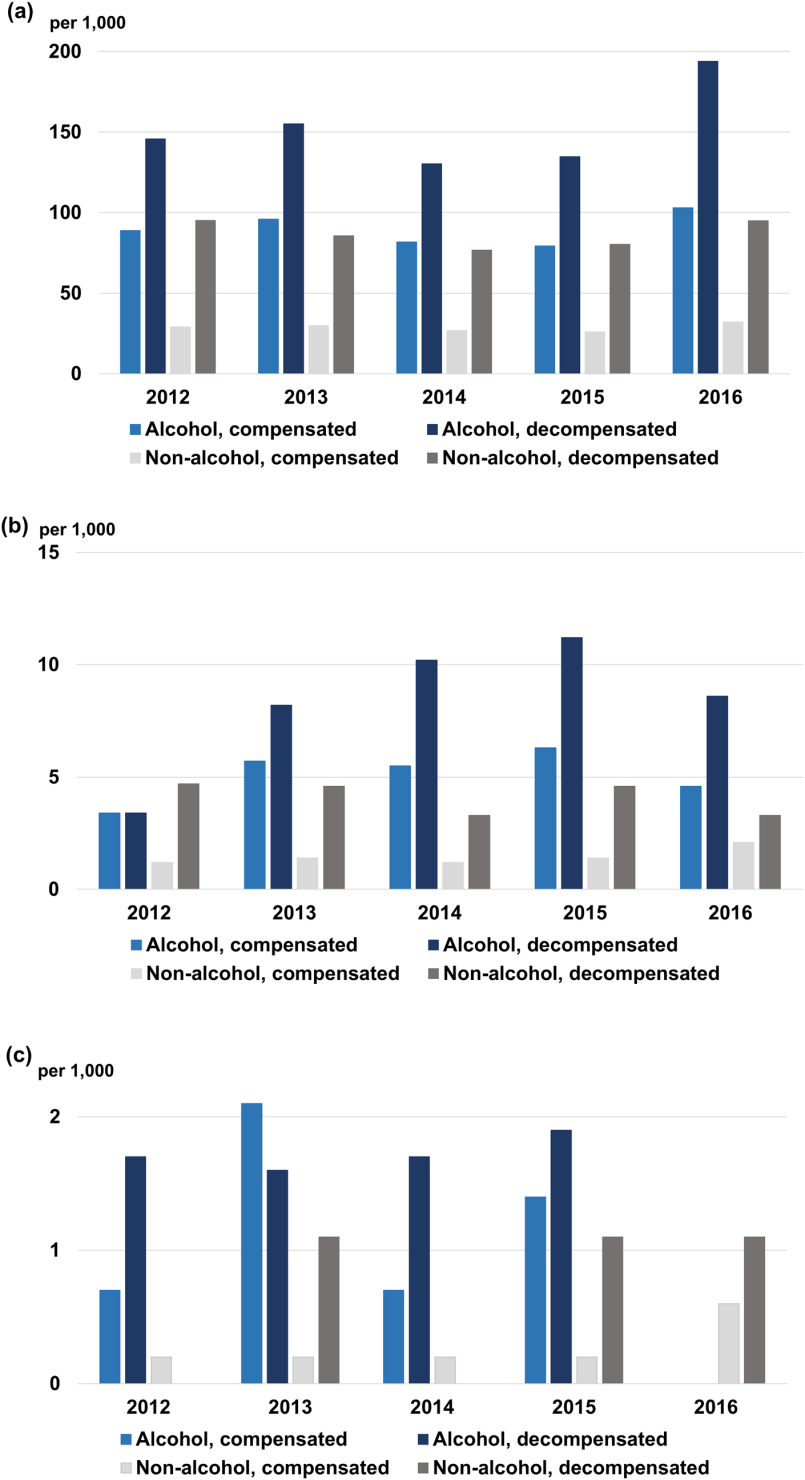
Incidence of critical events within 30 days of diagnosis (**a**) visiting an ED, (**b**) need for ICU care, and (**c**) death.

The proportion of patients who visited an ED within 30 days of their initial diagnosis was captured. For ALC, 79.4–103.1 per 1000 patients visited an ED. Patients with decompensated ALC visited an ED at the rate of 130.3–194.0 per 1000 patients (Table 2). Those proportions were 3.1–3.2 times higher than seen with non-alcoholic cirrhosis for compensated cirrhosis patients (by index year; 2012–2016, all *P* < 0.05), and 1.5–2.0 times higher than seen for decompensated cirrhosis patients (by index year; 2012–2016, all *P* < 0.05) (Fig. 3a; Supplementary Table S6). The proportion of ALD and ALC patients who visited an ED did not change significantly during the study period.

**Table 2:** Incidence rate of critical events related to liver cirrhosis by index year.

Critical events	2012	2013	2014	2015	2016	*P*
**Visit the emergency department within 30 days of diagnosis**						
Alcoholic cirrhosis, case/group, n	110/1238	114/1188	101/1234	98/1234	134/1300	
Proportion per 1000 patients	88.9	96.0	81.8	79.4	103.1	0.602
Alcoholic cirrhosis with decompensation, case/group, n	71/487	77/496	62/476	60/445	91/469	
Proportion per 1000 patients	145.8	155.2	130.3	134.8	194.0	0.144
Non-alcoholic cirrhosis, case/group, n	116/3997	123/4120	122/4558	118/4534	158/4916	
Proportion per 1000 patients	29.0	29.9	26.8	26.0	32.1	0.687
Non-alcoholic cirrhosis With decompensation, case/group, n	69/725	65/759	62/808	59/734	73/769	
Proportion per 1000 patients	95.2	85.6	76.7	80.4	94.9	0.887
**Need for intensive care unit care within 30 days of diagnosis**						
Alcoholic cirrhosis, case/group, n	5/1456	8/1408	8/1455	9/1422	7/1519	
Proportion per 1000 patients	3.4	5.7	5.5	6.3	4.6	0.620
Alcoholic cirrhosis with decompensation, case/group, n	2/592	5/612	6/591	6/537	5/583	
Proportion per 1000 patients	3.4	8.2	10.2	11.2	8.6	0.261
Non-alcoholic cirrhosis, case/group, n	5/4286	6/4408	6/4825	7/4841	11/5281	
Proportion per 1000 patients	1.2	1.4	1.2	1.4	2.1	0.260
Non-alcoholic cirrhosis With decompensation, case/group, n	4/843	4/869	3/917	4/866	3/903	
Proportion per 1000 patients	4.7	4.6	3.3	4.6	3.3	0.678
**Death within 30 days of diagnosis**						
Alcoholic cirrhosis, case/group, n	1/1463	3/1415	1/1458	2/1429	0/1530	
Proportion per 1000 patients	0.7	2.1	0.7	1.4	0	0.404
Alcoholic cirrhosis with decompensation, case/group, n	1/594	1/615	1/592	1/540	0/586	
Proportion per 1000 patients	1.7	1.6	1.7	1.9	0	0.508
Non-alcoholic cirrhosis, case/group, n	1/4289	1/4414	1/4834	1/4853	3/5297	
Proportion per 1000 patients	0.2	0.2	0.2	0.2	0.6	0.381
Non-alcoholic cirrhosis With decompensation, case/group, n	0/846	1/871	0/921	1/870	1/906	
Proportion per 1000 patients	0	1.1	0	1.1	1.1	0.431

The proportion of patients who needed ICU care within 30 days of their initial diagnosis was captured. For ALC, 3.4–6.3 per 1000 patients needed ICU care, and 3.4–11.2 per 1000 patients with decompensated cirrhosis needed ICU care (Table 2). The proportion of compensated cirrhosis patients who needed ICU care was 2.2–4.6 times higher than among non-alcoholic cirrhosis patients (by index year; 2012, *P* = 0.136; 2013, *P* = 0.004; 2014, *P* = 0.003; 2015, *P* = 0.004; and 2016, *P* = 0.091), and it was 0.7–2.6 times higher in decompensated cirrhosis patients than in non-alcoholic decompensated patients (by index year; 2012–2016, all *P* > 0.05) (Fig. 3b; Supplementary Table S6). The proportion of ALD and ALC patients who needed ICU care did not change significantly during the study period.

The proportion of patients who died within 30 days of their initial diagnosis was captured. For ALC, 0–2.1 per 1000 patients died, and the rate among patients with decompensated cirrhosis was 0–1.9 per 1000 patients (Table 2). With non-alcoholic cirrhosis, the proportion of compensated cirrhosis patients who died was 0.2–0.6 per 1000 patients (by index year; 2012, *P* = 0.444; 2013, *P* = 0.047; 2014, *P* = 0.410; 2015, *P* = 0.132; and 2016, *P* = 0.999), and the proportion of decompensated cirrhosis who died was 0–1.1 per 1000 patients (by index year; 2012–2016, all *P* > 0.05) (Fig. 3c; Supplementary Table S6). The proportion of ALD and ALC patients who died within 30 days of diagnosis did not change significantly during the study period.

### Management patterns after initial diagnosis of ARLD

**Figure 4:**
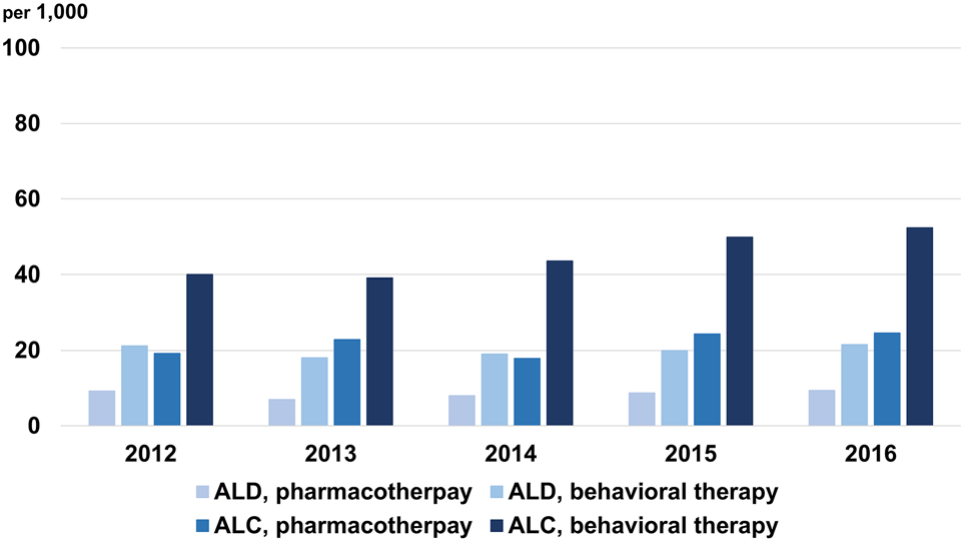
Initiation of pharmaco- or behavioral therapy within 60 days of an index diagnosis of alcohol-related liver disease.

We assessed the management patterns for ARLD by therapeutic method. For pharmacotherapy, 7.0–9.4 per 1000 ALD patients and 19.2–24.6 per 1000 ALC patients were treated within 60 days of diagnosis. For behavioral therapy, 18.1–21.6 per 1000 ALD patients and 39.1–52.4 per 1000 ALC patients were treated within 60 days of diagnosis (Table 3). About twice as many ALD and ALC patients received behavioral therapy compared with pharmacotherapy in the entire study period (2.3–2.6 times higher in ALD and 1.7–2.4 times higher in ALC, Fig. 4).

**Table 3:** Initiation of pharmacotherapy or behavioral therapy after an initial diagnosis of alcoholic liver disease or alcoholic liver cirrhosis by index year.

	2012	2013	2014	2015	2016
**Pharmacotherapy within 60 days of diagnosis**					
Alcoholic liver disease, case/group, n	132/14,244	98/13,990	110/13,727	127/14,433	135/14,434
Proportion per 1,000 patients	9.3	7.0	8.0	8.8	9.4
Alcoholic cirrhosis, case/group, n	27/1,406	31/1,353	25/1,397	33/1,352	36/1,466
Proportion per 1,000 patients	19.2	22.9	17.9	24.4	24.6
**Behavioral therapy within 60 days of diagnosis**					
Alcoholic liver disease, case/group, n	283/13,332	238/13,125	246/12,899	271/13,526	292/13,522
Proportion per 1,000 patients	21.2	18.1	19.1	20.0	21.6
Alcoholic cirrhosis, case/group, n	52/1,300	49/1,253	57/1,306	63/1,263	72/1,375
Proportion per 1,000 patients	40.0	39.1	43.6	49.9	52.4

* Pharmacotherapy: acamprosate, naltrexone.

**Figure 5:**
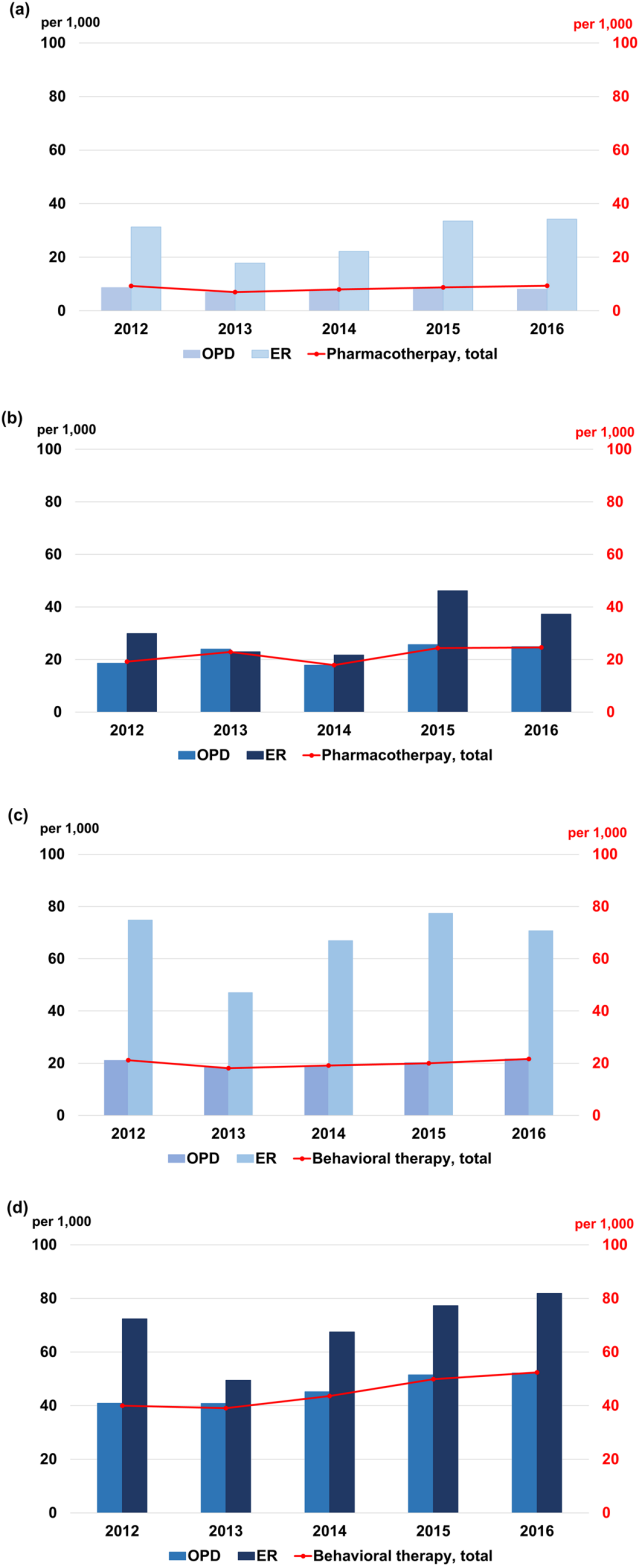
Pharmaco- or behavioral therapy for ARLD by the place of initial diagnosis. (**a**) ALD, pharmacotherapy; (**b**) ALC, pharmacotherapy; (**c**) ALD, behavioral therapy; (**d**) ALC, behavioral therapy.

The management patterns were assessed according to the place where the initial diagnosis was made, an outpatient department (OPD) or ED. ALD patients diagnosed in an ED were 2–4 times more likely than those diagnosed in an OPD to receive both pharmacotherapy (OPD, 7.0–8.7 per 1000 patients; ED, 17.8–34.2 per 1000 patients) and behavioral therapy (OPD, 18.3–21.6 per 1000 patients; ED, 47.0–77.4 per 1000 patients) (Table 4). ALC patients diagnosed in an ED also received more therapy than those diagnosed in an OPD, but the differences were smaller than among ALD patients in both pharmacotherapy (OPD, 17.9–24.9 per 1000 patients; ED, 21.7–46.2 per 1000 patients) and behavioral therapy (OPD, 40.8–52.2 per 1000 patients; ED, 49.5–81.9 per 1000 patients) (Table 4). Overall, the proportions of patients who received pharmacotherapy remained stationary during the study period for both ALD (< 2.5% at any visit, < 1.5% for all ALD) and ALC (< 3.0% at any visit, < 2.5% for all ALC). The initiation of behavioral therapy also remained stationary for both ALD (< 2.5% for all ALD) and ALC (< 5.5% for all ALC) (Fig. 5).

**Table 4:** Initiation of pharmacotherapy or behavioral therapy by the place alcoholic liver disease and alcoholic cirrhosis were first diagnosed.

	2012	2013	2014	2015	2016
**Alcoholic liver disease, case/group, n**					
Outpatient visit					
Pharmacotherapy within 60 days	122/14,027	97/13,781	104/13,534	124/14,213	114/14,039
Proportion per 1000 patients	8.7	7.0	7.7	8.7	8.1
Behavioral therapy within 60 days	278/13,178	238/12,978	241/12,740	269/13,344	288/13,333
Proportion per 1000 patients	21.1	18.3	18.9	20.2	21.6
Emergency department visit					
Pharmacotherapy within 60 days	40/1275	22/1234	27/1214	44/1312	50/1462
Proportion per 1000 patients	31.3	17.8	22.2	33.5	34.2
Behavioral therapy within 60 days	83/1110	51/1085	72/1076	89/1150	92/1301
Proportion per 1000 patients	74.8	47.0	66.9	77.4	70.7
**Alcoholic cirrhosis, case/group, n**					
Outpatient visit					
Pharmacotherapy within 60 days	25/1343	31/1294	24/1344	33/1285	35/1408
Proportion per 1000 patients	18.6	24.0	17.9	25.7	24.9
Behavioral therapy within 60 days	51/1248	49/1200	57/1261	62/1205	69/1322
Proportion per 1000 patients	40.9	40.8	45.2	51.5	52.2
Emergency department visit					
Pharmacotherapy within 60 days	14/469	10/434	10/460	20/433	19/510
Proportion per 1000 patients	29.9	23.0	21.7	46.2	37.3
Behavioral therapy within 60 days	31/428	20/404	28/415	31/401	39/476
Proportion per 1000 patients	72.4	49.5	67.5	77.3	81.9

*Pharmacotherapy: acamprosate, naltrexone.

## Discussion

In this nationwide, population-based study, we evaluated the incidence and management patterns for ARLD using five years of data from the well-established HIRA-NPS database. Although some previous studies had attempted to capture the status of alcohol consumption and prevalence of ARLD^9,10,31^, no previous study compared the incidence of ARLD with that of other liver disease etiologies in Korea. Furthermore, this study is the first attempt to capture the real-world management patterns for ARLD. Our most important finding is that the incidence rate of ARLD has not decreased, and the proportion of cirrhosis at diagnosis was more than twice as high among ARLD patients than among those with another liver disease etiology. Unfortunately, the initiation of ARLD management was low regardless of the therapeutic method chosen or the department in which the diagnosis was made. Previous studies have shown that most patients with problematic drinking are unaware of their liver disease until they suffer certain symptoms^32,33^. Therefore, even this nationwide data might underestimate both the disease burden and the initiation of alcohol cessation management for ARLD. Generosity about alcohol-related problems in Korean culture could mean that many ARLD patients remain undetected^10^. Cessation of alcohol use is the most effective way to reduce the burden of ARLD^11,34^, and our study indicates that ARLD management still needs more attention.

A recent report showed that the nationwide burden from alcohol use had changed and that the proportion of young adults and females among risky alcohol users had increased^10^. Our data show a similar trend for females, but the age distribution of ARLD during our study period became older, and the comorbidity index of patients increased. Although we could not analyze patient-level data, the real-world burden of advanced ARLD appears to have shifted toward elderly patients with more comorbidities compared with earlier studies. There have been several population-based cohort studies analyzing ARLD. One cohort study from the United States demonstrated that the prevalence of ARLD among elderly, female, and ALC subjects was increased, but without statistical significance^35^. Another incidence-based trend analysis showed that the ALC group becomes older between 2004 and 2014^36^. One multinational study suggested that ARLD patients were younger and had more comorbidities than HCV patients^37^. Overall, these epidemiologic trends of ARLD are similar to those reported from other countries with high alcohol consumption^5,35^. Along the same lines, ARLD remains a major contributor to the cirrhotic burden, with a higher proportion of decompensated patients than in non-ARLD, which suggests that uncontrolled drinking leads to disease progression and acute exacerbation in Korea. Furthermore, ALC patients had a higher CCI than ALD patients, which suggests that more vulnerable patients were suffering more severe ARLD, and that finding did not change during our study period.

The higher proportion of decompensated cirrhosis raises concerns about the actual burden of ARLD. To evaluate the disease burden of ARLD, we focused on patients initially diagnosed with ALC compared to those with non-alcoholic cirrhosis. We evaluated the incidence of three events within 30 days of initial diagnosis to indicate the severity of cirrhosis: ED visit, ICU care, and death^24–28^. ALC showed higher decompensation than non-alcoholic cirrhosis, as shown by the three severity indicators of short-term prognosis. These results are consistent with those of previous studies in other countries^38–41^, which suggests that the incidence of severe manifestations could reflect that ARLD patients face poor management and later diagnosis than patients with other etiologies for liver disease.

In terms of assessing the management patterns, we evaluated the initiation of management for alcohol use cessation without considering acute phase therapies such as glucocorticoid challenge for severe alcoholic hepatitis or detoxification therapy for withdrawal syndrome^2^. For the cessation of alcohol use, both pharmacotherapy and behavioral therapy are important^11,34^. Therefore, we attempted to measure how physicians treat ARLD after the initial diagnosis. No study has examined how many patients need to start cessation therapy after being diagnosed with ARLD. Considering the general prevalence of high-risk drinking, 10–20% of ARLD patients could need to initiate management regardless of severity^1,42^. Unfortunately, the rate of management was < 10% regardless of severity or place of diagnosis, even among patients diagnosed with ALC. Intriguingly, ARLD patients diagnosed at an ED had a higher chance of entering management than patients diagnosed at an OPD. That trend was more prominent in ALC patients, who already had progressive disease, than ALD patients. These results suggest that management is mainly provided for severe forms of ARLD, not to prevent disease progression. Further study is needed to determine what factors affect management patterns for ARLD treatment in Korea.

Our study has several limitations. We used data from HIRA-NPS, an administrative claims-based database, and that made it difficult to assess detailed clinical information and impossible to assess data not collected by the insurance program. The actual incidence of ARLD could be higher than what we captured in this study because many ARLD patients had low socioeconomic status, which correlates with poor adherence to medical care standards^1,7^. In addition, most cessation management, such as psychoactive drugs and psychiatric interventions, were provided under insurance reimbursement, which could be an additional confounder. Overall, the real-world proportion of patients who receive management from among those who need it could be lower than our study indicates. We captured the initiation of management within 60 days of diagnosis. No previous study has evaluated the optimal interval for initiating cessation management. However, some intervals may be needed to treat alcoholic hepatitis or detoxification. The interval in previous studies varied from 7 to 30 days after diagnosis^1,32,43–45^. The proper interval for starting cessation management needs further study. Also, we could not evaluate the safety and efficacy of pharmacotherapy in our study, especially among ALC patients^2^, and the definition of behavioral therapy we used could not distinguish the psychiatric condition targeted. However, the initiation of behavioral therapy within a short interval could reflect various psychiatric conditions related to ARLD so that it should be included as a target population of study purposes^46,47^. Overall, a future study needs to elucidate the best way to manage ARLD in terms of initiation timing, therapeutic combination, safety, and efficacy.

In conclusion, ARLD still accounts for a major portion of liver disease, manifesting in a more severe form than liver diseases arising from other etiologies in Korea. Furthermore, patients more vulnerable to alcohol suffered more severe ARLD. Unfortunately, the initial management for alcohol use cessation was very low in the study period. Physicians should pay more attention to the management of ARLD, identify patients with suitable treatment indications, and monitor whether appropriate treatment is applied.

## Supplementary Information

Supplementary Information 1.

Supplementary Information 2.
