# Supplementary material for: Incidence and management patterns of alcohol-related liver disease in Korea: a nationwide standard cohort study
Source: Sci Rep. 2021 Mar 23;11:6648. doi: 10.1038/s41598-021-86197-z (PMC7987970; doi:10.1038/s41598-021-86197-z)
Supplement: Supplementary file 1 — Supplementary Information 1. [file 41598_2021_86197_MOESM1_ESM.docx]

**Title**: Incidence and management patterns of alcohol-related liver disease in Korea: A nationwide standard cohort study

**Short title:** New strategies needed for alcohol-related liver disease in Korea

Ha Il Kim,^1^ Seo Young Park,^2^ Hyun Phil Shin^1,3*^

^1^Division of Gastroenterology, Department of Internal Medicine, Kyung Hee University Hospital at Gangdong, Seoul, Korea

^2^Department of Clinical Epidemiology and Biostatistics, Asan Medical Center, Seoul, Korea

^3^Division of Gastroenterology, Department of Internal Medicine, Kyung Hee University School of Medicine, Seoul, Korea

**Supplementary table 1**. Concept identification and concept codes for concept sets

| Concept sets | | Concept identification | Concept code | Name |
| --- | --- | --- | --- | --- |
| Alcohol-related disorder | | 36714559, 318773, 378421, 195300, 376383, 4340964, 4340493, 4078688, 201612, 4340383, 4340386, 193256, 196463, 4340385 | 719848005, 83521008, 7916009, 2043009, 29212009, 235952002, 235942001, 19303008, 41309000, 235875008, 235881000, 50325005, 420054005, 235880004 | Disorder caused by alcohol, Dilated cardiomyopathy secondary to alcohol, Alcoholic polyneuropathy, Alcoholic gastritis, Alcohol-induced organic mental disorder, Alcohol-induced chronic pancreatitis, Alcohol-induced acute pancreatitis, Alcohol myopathy, Alcoholic liver damage, Alcoholic hepatitis, Alcoholic hepatic failure, Alcoholic fatty liver, Alcoholic cirrhosis, Alcoholic fibrosis and sclerosis of liver |
| Alcohol-related liver disease | Alcoholic liver disease | 201612, 4340383, 4340386, 193256 | 41309000, 235875008, 235881000, 50325005 | Alcoholic liver damage, Alcoholic hepatitis, Alcoholic hepatic failure, Alcoholic fatty liver |
|  | Alcoholic liver cirrhosis | 196463, 4340385 | 420054005, 235880004 | Alcoholic cirrhosis, Alcoholic fibrosis and sclerosis of liver |
| Liver cirrhosis | | 4064161, 4058695, 4046123, 4340394, 4340948, 4267417, 192675, 4340385, 196463 | 19943007, 197362001, 12368000, 235899008, 235901004, 62484002, 1761006, 235880004, 420054005 | Cirrhosis of liver, Toxic liver disease with fibrosis and cirrhosis of liver, Secondary biliary cirrhosis, Hepatic sclerosis, Hepatic fibrosis with hepatic sclerosis, Hepatic fibrosis, Biliary cirrhosis, Alcoholic fibrosis and sclerosis of liver, Alcoholic cirrhosis |
| Features of decompensated cirrhosis | | 4029488, 196029, 377604, 4237824, 2108892, 22340, 28779, 200528 | 139200009, 40946000, 72836002, 91109007, 43244, 14223005, 17709002, 389026000 | Hepatic encephalopathy, Hepatic coma due to viral hepatitis, Hepatic coma, Gastric varices, Band ligation of esophageal/gastric varices, Esophageal varices without bleeding, Bleeding esophageal varices, Ascites |
| Chronic viral hepatitis | | 439674, 192240, 197493, 198964, 4012113 | 186639003, 235869004, 235865005, 128302006, 10295004 | Chronic viral hepatitis B without delta-agent, Hepatitis D superinfection of hepatitis B carrier, Chronic viral hepatitis B with hepatitis D, Chronic hepatitis C, Chronic viral hepatitis |
| Non-alcoholic fatty liver disease* | | 4026131, 4059290, 40484532 | 197315008, 197321007, 442685003 | Non-alcoholic fatty liver, Steatosis of liver*, Nonalcoholic steatohepatitis |
| Minor etiologies for chronic liver disease | | 4135822,40577758,  4229262, 4163735,200762 | 31712002,4032000,  88518009,  399187006, 408335007 | Primary biliary cholangitis, Primary sclerosing cholangitis, Wilson’s disease, Hemochromatosis, Autoimmune hepatitis |
| Primary liver cancer | | 4001171 | 109841003 | Liver cell carcinoma |
| Liver transplantation | | 42537742 | 737297006 | Transplanted liver present |
| Death | | 4249566, 4052310, 4306655 | 409651001, 16100001, 419620001 | Mortality rate, Death diagnosis, Death |
| Pharmacotherapy | | 1714316, 44787878, 19043959, 36881894 | 7243, 1483744, 82819, OMOP988607 | Naltrexone, Naltrexone oral Tablet, Acamprosate, Acamprosate oral Tablet |
| Behavioral therapy | | 4088889, 4028920, 4296166 | 18512000, 108313002, 76168009 | Individual psychotherapy, Family psychotherapy procedure, Group psychotherapy |
| Intensive care unit | | 4335583, 4074665, 4051329, 4050863 | 232677006, 243147009, 233580005, 233578004 | Tracheal intubation, controlled ventilation, Continuous venovenous hemodialysis, Continuous hemodialysis |
|  |  | 1321341,19011871, 1507835, 1507838, 1337860, 1343916, 19076899 | 7512, 142104, 11149, 313578, 3628, 3992, 310132 | Norepinephrine, Norepinephrine injectable solution, Vasopressin, Vasopressin injectable solution, Dopamine, Epinephrine, Epinephrine injectable solution |
| Visit occurrence | | 9203, 262, 9202, 9201 | ER, ERIP, OP, IP | Emergency room visit, Emergency room and Inpatient visit, Outpatient visit, Inpatient visit |

Charlson comorbidity index: Preset ID 49, Domain: CONDITION, design: Charlson Index, Description: “The Charlson comorbidity index (Romano adaptation) using all conditions prior to the window end” [5-7]; * Steatosis of liver: excludes concept sets of “Alcoholic related disease” and “Alcoholic liver disease”

**Supplementary table 2.** Demographic characteristics of the total study population by index year

| Demographic characteristics | 2012 | 2013 | 2014 | 2015 | 2016 | *P* |
| --- | --- | --- | --- | --- | --- | --- |
| **Total study population** | 1,421,700 | 1,430,800 | 1,421,300 | 1,453,500 | 1,468,000 |  |
| **Alcohol-related disorders** | 18,989 | 18,882 | 18,658 | 19,702 | 19,573 |  |
| Proportion per 100,000 people | 1,336 | 1,320 | 1,313 | 1,356 | 1,333 |  |
| Age, m ± SD | 51.97 (13.16) | 52.46 (13.19) | 50.79 (13.08) | 53.03 (13.64) | 53.41 (13.53) | <0.001 |
| Age group, n (%) |  |  |  |  |  | <0.001 |
| < 50 yr | 7,803 (41.1) | 7,432 (39.4) | 8,341 (44.7) | 7,494 (38.0) | 7,188 (36.7) |  |
| ≥ 50 yr | 11,186 (58.9) | 11,450 (60.6) | 10,317 (55.3) | 12,208 (62.0) | 12,385 (63.3) |  |
| Sex, n (%) |  |  |  |  |  | <0.001 |
| Male | 14,752 (77.7) | 14,668 (77.7) | 14,336 (76.8) | 14,956 (75.9) | 14,709 (75.1) |  |
| Female | 4,237 (22.3) | 4,214 (22.3) | 4,322 (23.2) | 4,746 (24.1) | 4,864 (24.9) |  |
| Charlson comorbidity index, m ± SD | 1.46 ± 1.90 | 1.47 ± 1.88 | 1.53 ± 1.96 | 1.58 ± 1.97 | 1.60 ± 1.94 | <0.001 |
| **Alcohol-related**  **Gastrointestinal disorders** | 15,522 | 15,232 | 14,993 | 15,746 | 15,639 |  |
| Age, m ± SD | 52.52 (12.98) | 52.89 (12.98) | 51.09 (12.88) | 53.53 (13.42) | 54.07 (13.22) | <0.001 |
| Age group, n (%) |  |  |  |  |  | <0.001 |
| < 50 yr | 6,152 (39.6) | 5,766 (37.9) | 6,545 (43.7) | 5,724 (36.4) | 5,426 (34.7) |  |
| ≥ 50 yr | 9,370 (60.4) | 9,466 (62.1) | 8,448 (56.3) | 10,019 (63.6) | 10,213 (65.3) |  |
| Sex, n (%) |  |  |  |  |  | <0.001 |
| Male | 12,158 (78.3) | 11,878 (78.0) | 11,569 (77.1) | 11,979 (76.1) | 11,783 (75.3) |  |
| Female | 3,364 (21.7) | 3,354 (22.0) | 3,424 (22.8) | 3,764 (23.9) | 3,856 (24.7) |  |
| Charlson comorbidity index, m ± SD | 1.64 (1.94) | 1.66 (1.92) | 1.74 (1.98) | 1.79 (2.01) | 1.82 (1.92) | <0.001 |
| **Alcohol-related**  **Neuropsychiatric disorders** | 798 | 718 | 837 | 835 | 784 |  |
| Age, m ± SD | 56.67 ± 12.90 | 56.99 ± 11.82 | 54.30 ± 12.37 | 57.05 ± 12.05 | 58.23 ± 11.97 | <0.001 |
| Sex, n (%) |  |  |  |  |  | 0.002 |
| Male | 693 (86.8) | 638 (88.9) | 732 (87.5) | 717 (85.9) | 644 (82.1) |  |
| Female | 105 (13.2) | 80 (11.1) | 105 (12.5) | 118 (14.1) | 140 (17.9) |  |
| Charlson comorbidity index, m ± SD | 1.71 ± 2.12 | 1.66 ± 2.02 | 1.86 ± 2.06 | 2.04 ± 2.25 | 2.08 ± 2.51 | 0.008 |
| **Alcohol-related cardiac disorders** | 16 | 23 | 21 | 21 | 21 |  |
| Age, (min-max) | 49.88 (28-80) | 53.78 (35-72) | 45.52 (30-66) | 55.96 (40-82) | 52.38 (33-69) |  |
| Sex, n (%) |  |  |  |  |  |  |
| Male | 15 (93.8) | 22 (95.7) | 20 (95.2) | 19 (90.5) | 21 (100) |  |
| Female | 1 (6.2) | 1 (4.3) | 1 (4.8) | 2 (9.5) | 0 (0) |  |

**Supplementary table 3.** Comparison of the general characteristics of the study population between alcoholic liver disease and alcoholic liver cirrhosis by index year

|  | 2012 | | | 2013 | | | | | 2014 | | | | 2015 | | | | 2016 | | |
| --- | --- | --- | --- | --- | --- | --- | --- | --- | --- | --- | --- | --- | --- | --- | --- | --- | --- | --- | --- |
|  | ALD  N=14,561 | ALC  N=1,463 | *P* | | ALD  N=14,327 | ALC  N=1,415 | *P* | ALD  N=14,061 | | ALC  N=1,458 | *P* | ALD  N=14,832 | | ALC  N=1,429 | *P* | ALD  N=14,775 | | ALC  N=1,530 | *P* |
| Age,  m ± SD [95% CI] | 52.62  ±12.78  [52.41-52.83] | 56.78  ±11.08  [56.21-57.35] | <0.001 | | 53.07  ± 12.82  [52.86-53.28] | 57.33  ±10.94  [56.76-57.90] | <0.001 | 51.32  ±12.64  [51.11-51.53] | | 55.55  ±11.41  [54.96-56.14] | <0.001 | 53.81  ±13.16  [53.60-54.02] | | 58.00  ±11.19  [57.42-58.58] | <0.001 | 54.30  ±12.99  [54.09-54.51] | | 57.62  ±11.02  [57.07-58.17] | <0.001 |
| Male,  n (%)  [95% CI] | 11,498 (79.0)  [0.78-0.80] | 1,268 (86.7)  [0.85-0.88] | <0.001 | | 11,197 (78.2)  [0.77-0.79] | 1,237 (87.4)  [0.86-0.89] | <0.001 | 10,925 (77.7)  [0.77-0.78] | | 1,268 (87.0)  [0.85-0.87] | <0.001 | 11,408 (76.9)  [0.76-0.76] | | 1,207 (84.5)  [0.82-0.86] | <0.001 | 11,219 (75.9)  [0.75-0.77] | | 1,307 (85.4)  [0.84-0.87] | <0.001 |
| CCI,  m ± SD  [95% CI] | 1.39  ±1.95  [1.36-1.42] | 3.42  ±2.45  [3.29-3.55] | <0.001 | | 1.70  ±1.93  [1.67-1.73] | 3.45  ±2.48  [3.32-3.58] | <0.001 | 1.75  ±1.99  [1.72-1.78] | | 3.50  ±2.46  [3.37-3.63] | <0.001 | 1.83  ±2.00  [1.80-1.86] | | 3.51  ±2.46  [3.38-3.64] | <0.001 | 1.86  ±1.97  [1.83-1.89] | | 3.47  ±2.38  [3.35-3.59] | <0.001 |

ALD, alcoholic liver disease; ALC, alcoholic liver cirrhosis; m, mean; SD, standard deviation; CCI, Charlson comorbidity index; CI, confidence interval

**Supplementary table 4.** Comparison of the general characteristics of the study population between alcoholic liver cirrhosis and non-alcoholic liver cirrhosis by index year

|  | 2012 | | | 2013 | | | | | 2014 | | | | 2015 | | | | 2016 | | |
| --- | --- | --- | --- | --- | --- | --- | --- | --- | --- | --- | --- | --- | --- | --- | --- | --- | --- | --- | --- |
|  | ALC  N=1,463 | NonALC  N=4,289 | *P* | | ALC  N=1,415 | NonALC  N=4,414 | *P* | ALC  N=1,458 | | NonALC  N=4,834 | *P* | ALC  N=1,429 | | NonALC  N=4,853 | *P* | ALC  N=1,530 | | NonALC  N=5,298 | *P* |
| Age,  m ± SD [95% CI] | 52.78  ±11.08  [56.21-57.34] | 57.15  ±12.27  [56.78-57.52] | <0.001 | | 57.33  ±10.94  [56.76-57.90] | 57.86  ±12.42  [57.49-58.22] | <0.001 | 55.55  ±11.41  [54.96-56.14] | | 56.02  ±12.29  [55.67-56.37] | <0.001 | 58.00  ±11.19  [57.42-58.58] | | 58.31  ±12.29  [57.96-58.66] | <0.001 | 57.62  ±11.02  [57.07-58.17] | | 58.99  ±12.62  [58.65-59.33] | <0.001 |
| Male,  n (%)  [95% CI] | 1,268 (86.7)  [0.85-0.88] | 2,652 (61.8)  [0.60-0.63] | <0.001 | | 1,237 (87.4)  [0.86-0.89] | 2,718 (61.6)  [0.60-0.63] | <0.001 | 1,268 (87.0)  [0.85-0.89] | | 3,019 (62.5)  [0.61-0.64] | <0.001 | 1,207 (84.5)  [0.82-0.86] | | 3,009 (62.0)  [0.61-0.63] | <0.001 | 1,307 (85.4)  [0.84-0.87] | | 3,187 (60.2)  [0.59-0.61] | <0.001 |
| CCI,  m ± SD  [95% CI] | 3.42  ±2.45  [3.29-3.55] | 2.93  ±2.28  [2.86-3.00] | <0.001 | | 3.45  ±2.48  [3.32-3.58] | 3.00  ±2.31  [2.93-3.07] | <0.001 | 3.50  ±2.46  [3.37-3.63] | | 3.05  ±2.35  [2.98-3.12] | <0.001 | 3.51  ±2.46  [3.38-3.64] | | 3.07  ±2.33  [3.00-3.14] | <0.001 | 3.47  ±2.38  [3.35-3.59] | | 3.16  ±2.42  [3.09-3.22] | <0.001 |

ALC, alcoholic liver cirrhosis; NonALC, non-alcoholic liver cirrhosis, m, mean; SD, standard deviation; CCI, Charlson comorbidity index; CI, confidence interval

**Supplementary table 5.** Demographic characteristics of liver cirrhosis in the study population by index year

| Demographic characteristics | 2012 | 2013 | 2014 | 2015 | 2016 |
| --- | --- | --- | --- | --- | --- |
| **Total liver cirrhosis cases** | 6,138 | 6,191 | 6,640 | 6,730 | 7,268 |
| **Alcoholic liver cirrhosis**  **with other liver disease** | 386 | 362 | 348 | 448 | 440 |
| **Liver cirrhosis**  **without alcohol-related disease** | 4,289 | 4,414 | 4,834 | 4,853 | 5,298 |
| Chronic viral hepatitis, n (%) | 2,637 (61.5) | 2,765 (62.6) | 3,154 (65.2) | 3,146 (64.8) | 3,016 (56.9) |
| Other, n (%) | 1,652 (38.5) | 1,649 (37.4) | 1,680 (34.8) | 1,707 (35.2) | 2,282 (43.1) |
| Age, m ± SD | 57.15 ± 12.27 | 57.86 ± 12.42 | 56.02 ± 12.29 | 58.31 ± 12.29 | 58.99 ± 12.62 |
| Sex, n (%) |  |  |  |  |  |
| Male | 2,652 (61.8) | 2,718 (61.6) | 3,019 (62.5) | 3,009 (62.0) | 3,187 (60.2) |
| Charlson comorbidity index, m ± SD | 2.93 ± 2.28 | 3.00 ± 2.31 | 3.05 ± 2.35 | 3.07 ± 2.33 | 3.16 ± 2.42 |
| Decompensation at diagnosis, n (%) | 846 (19.7) | 871 (19.7) | 921 (19.1) | 870 (18.0) | 906 (17.1) |

**Supplementary table 6.** Comparison of the incidence rate of critical events* between alcoholic and non-alcoholic liver cirrhosis by index year

|  |  | 2012 | | | 2013 | | | 2014 | | | | 2015 | | | | 2016 | | |
| --- | --- | --- | --- | --- | --- | --- | --- | --- | --- | --- | --- | --- | --- | --- | --- | --- | --- | --- |
|  |  | ALC | NonALC | *P* | ALC | NonALC | *P* | ALC | NonALC | *P* | ALC | | NonALC | *P* | ALC | | NonALC | *P* |
| LC-C  (case/  group, n) | Visit ED | 110/  1,238 | 116/  3,997 | <0.001 | 114/  1,188 | 123/  4,120 | <0.001 | 101/  1,234 | 122/  4,558 | <0.001 | 98/  1,234 | | 118/  4,534 | <0.001 | 134/  1,300 | | 158/  4,916 | <0.001 |
|  | Need  ICU | 5/  1,456 | 5/  4,286 | 0.136 | 8/  1,408 | 6/  4,408 | 0.004 | 8/  1,455 | 6/  4,825 | 0.003 | 9/  1,422 | | 7/  4,841 | 0.004 | 7/  1,519 | | 11/  5,281 | 0.091 |
|  | Death | 1/  1,463 | 1/  4,289 | 0.444 | 3/  1,415 | 1/  4,414 | 0.047 | 1/  1,458 | 1/  4,834 | 0.410 | 2/  1,429 | | 1/  4,853 | 0.132 | 0/  1,530 | | 3/  5,297 | 0.999 |
| LC-D  (case/  group, n)) | Visit ED | 71/  487 | 69/  725 | 0.007 | 77/  496 | 65/  759 | <0.001 | 62/  476 | 62/  808 | 0.002 | 60/  445 | | 59/  734 | 0.003 | 91/  469 | | 73/  769 | <0.001 |
|  | Need  ICU | 2/  592 | 4/  843 | 0.999 | 5/  612 | 4/  869 | 0.501 | 6/  591 | 3/  917 | 0.167 | 6/  537 | | 4/  866 | 0.195 | 5/  583 | | 3/  903 | 0.275 |
|  | Death | 1/  594 | 0/  846 | 0.413 | 1/  615 | 1/  871 | 0.999 | 1/  592 | 0/  921 | 0.391 | 1/  540 | | 1/  870 | 0.999 | 0/  586 | | 1/  906 | 0.999 |

LC-C, compensated liver cirrhosis; LC-D, decompensated liver cirrhosis; ALC, alcoholic liver cirrhosis; NonALC, non-alcoholic liver cirrhosis, ED, emergency department; ICU, intensive care unit, *Critical events: visit ED (visit the emergency department within 30 days of diagnosis), Need ICU (Need for intensive care unit care within 30 days of diagnosis), Death (Death within 30 days of diagnosis)
