# Supplementary figures and images for: Incidence and management patterns of alcohol-related liver disease in Korea: a nationwide standard cohort study
Source: Sci Rep. 2021 Mar 23;11:6648. doi: 10.1038/s41598-021-86197-z (PMC7987970; doi:10.1038/s41598-021-86197-z)

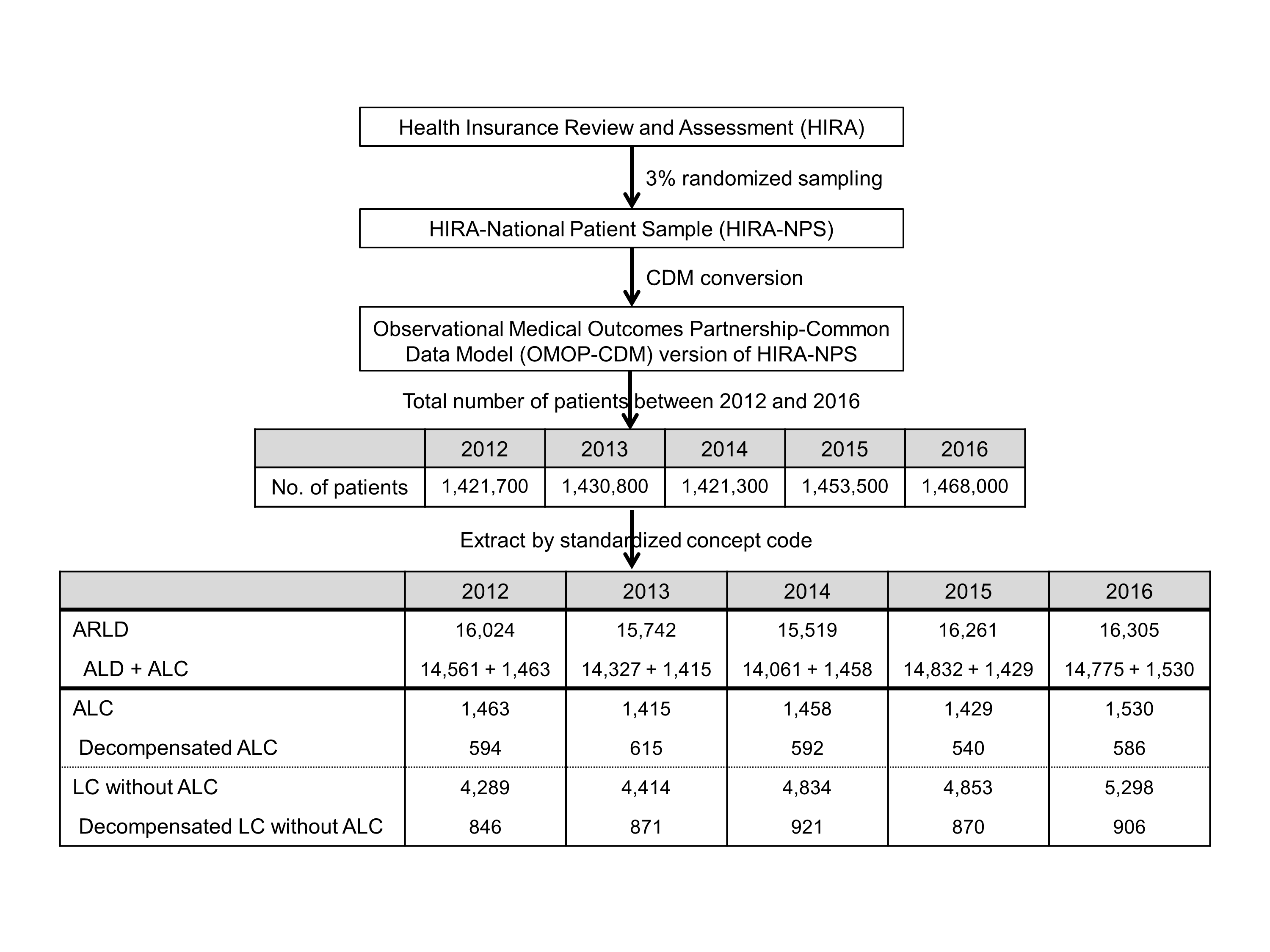

Supplement: Supplementary file 2 — Supplementary Information 2. [file 41598_2021_86197_MOESM2_ESM.tif]
